# Functional Roles of Chemokine Receptor CCR2 and Its Ligands in Liver Disease

**DOI:** 10.3389/fimmu.2022.812431

**Published:** 2022-02-25

**Authors:** Shaoping She, Liying Ren, Pu Chen, Mingyang Wang, Dongbo Chen, Ying Wang, Hongsong Chen

**Affiliations:** ^1^ Peking University Hepatology Institute, Beijing Key Laboratory of Hepatitis C and Immunotherapy for Liver Diseases, Peking University People’s Hospital, Beijing, China; ^2^ Laboratory of Hepatobiliary and Pancreatic Surgery, Affiliated Hospital of Guilin Medical University, Guilin, China; ^3^ State Key Laboratory of Experimental Hematology, National Clinical Research Center for Blood Diseases, Haihe Laboratory of Cell Ecosystem, Institute of Hematology & Blood Diseases Hospital, Chinese Academy of Medical Sciences & Peking Union Medical College, Tianjin, China; ^4^ Department of Immunology, School of Basic Medical Sciences, and NHC Key Laboratory of Medical Immunology, Peking University, Beijing, China

**Keywords:** chemokine, CCR2, CCL2, PSMP, hepatocellular carcinoma, macrophage

## Abstract

Chemokines are a family of cytokines that orchestrate the migration and positioning of immune cells within tissues and are critical for the function of the immune system. CCR2 participates in liver pathology, including acute liver injury, chronic hepatitis, fibrosis/cirrhosis, and tumor progression, by mediating the recruitment of immune cells to inflammation and tumor sites. Although a variety of chemokines have been well studied in various diseases, there is no comprehensive review presenting the roles of all known chemokine ligands of CCR2 (CCL2, CCL7, CCL8, CCL12, CCL13, CCL16, and PSMP) in liver disease, and this review aims to fill this gap. The introduction of each chemokine includes its discovery, its corresponding chemotactic receptors, physiological functions and roles in inflammation and tumors, and its impact on different immune cell subgroups.

## Introduction

Chemokines are a large family of small, highly conserved secretory proteins composed of nearly 50 cytokines (1). Chemokines were initially defined based on their ability to induce directed migration of immune cells to the inflammation or tumor microenvironment sites by binding to seven-transmembrane domain G protein-coupled receptors (GPCRs) (1, 2). According to the number of conserved cysteines present in their amino acid sequences, chemokines can be divided in four subfamilies (CXC, CC, C, and CX3C) (1, 3). Correspondingly, there are currently nearly 20 chemokine receptors, named CXCR, CCR, XCR, and CX3CR, according to the predominant types of chemokines bound (3, 4). The binding of chemokines to their receptors triggers intracellular signaling events, thereby promoting subsequent signaling cascades and leading to chemotaxis, degranulation, actin rearrangement, and release of superoxide anions (2, 3). C-C motif chemokine receptor 2 (CCR2) is a functional chemotactic receptor primarily expressed on the surface of monocytes/macrophages and lymphocytes, regulating many diseases by controlling the bone marrow monocytes’ mobilization into the bloodstream and their migration to inflammatory sites (4, 5). In the liver, CCR2 is involved in multiple stages of liver pathology, including acute liver injury and chronic hepatitis, fibrosis/cirrhosis, and tumor progression, making it a potential therapeutic target for hepatocellular carcinoma (HCC) (6–8). CCR2 can promote liver fibrosis by regulating the migration of circulating monocytes to the injured liver and through activation of hepatic stellate cells (HSCs) (7, 8). In addition, both preclinical and clinical studies have shown that a dual CCR2/CCR5 antagonist, cenicriviroc (CVC), is an effective and safe antifibrotic agent for treating nonalcoholic steatohepatitis (NASH) and alcohol-induced steatohepatitis (9–12). In the tumor microenvironment, knockout of CCR2 or intervention with CCR2 antagonists can inhibit HCC tumor growth and metastasis by regulating the recruitment and polarization of tumor-associated macrophages (TAMs) and can improve survival in mouse HCC models (13).

A remarkable feature of chemokines is redundancy and lack of specificity in the binding of chemokine receptors; that is, one chemokine can bind to several chemokine receptors, and one chemokine receptor can also bind to several chemokines (3, 4). For example, as we summarize in **Table 1**, CCR2 is not only a high-affinity receptor for members of the monocyte chemotactic protein (MCP) family, including C-C motif chemokine ligand 2 (CCL2), CCL7, CCL8, CCL12 (mouse only), and CCL13 (human only) (33–37). CCR2 can also bind to CCL16 and PC3-secreted microprotein (PSMP) or microseminoprotein (MSMP) with high affinity (38–40). PSMP is a novel chemotactic cytokine and functions as a high-affinity ligand for CCR2 according to Ying Wang’s laboratory findings (40). The full-length mature peptide of PSMP has 103 amino acids, including ten cysteines, which have both CC structure and CXC structure, unlike the characteristic sequences contained in classical chemokine structures (40).

**Table 1:** Relevant chemokine ligands of CCR2 in the pathogenesis of liver diseases.

Chemokine	Alternative name(s)	Reported chemokine receptor(s)	Responding cell type(s)	Involvement in liver disease	Functions	Refs
CCL2	MCP-1	CCR1, CCR2, CCR4, CCR11	Monocyte/Macrophage, Neutrophil, T cell, HSC, HCC cell	Acute liver injury, ALD, HBV/HCV, NAFLD/NASH, Fibrosis/Cirrhosis, HCC	Recruitment of monocytes/macrophages; Activation of KCs; Apoptosis resistance; Promotes HCC cell migration, invasion, EMT; Promotes tumor angiogenesis, metastasis.	(13–23)
CCL7	MCP-3	CCR1, CCR2, CCR3, CCR5	Monocyte/Macrophage, HCC cell	Acute liver injury, NASH, HCC	Recruitment of monocytes/macrophages; Promotes HCC cell migration, invasion, EMT.	(24–26)
CCL8	MCP-2	CCR1, CCR2, CCR3, CCR5, CCR11	Monocyte/Macrophage, T cell	HCV, NASH	–	(27–29)
CCL12	MCP-5	CCR2	Monocyte/Macrophage, T cell	NAFLD, HCC	–	(30)
CCL13	MCP-4	CCR1, CCR2, CCR3, CCR5, CCR11	Monocyte/Macrophage, eosinophil	–	–	–
CCL16	LEC, HCC-4, LMC, LCC-1	CCR1, CCR2, CCR5	Macrophage, T cell, HSC	Fibrosis/ Cirrhosis	Inactivation of HSCs.	(31)
PSMP	MSMP	CCR2	Monocyte/Macrophage, HSC	Acute liver injury, Fibrosis/ Cirrhosis, HCC	Recruitment of monocytes/macrophages; Activation of HSCs.	(32)

ALD, alcoholic liver disease; EMT, epithelial-mesenchymal transition; HBV, hepatitis B virus; HCV, hepatitis C virus; HCC, hepatocellular carcinoma; HSCs, hepatic stellate cells; KC, Kupffer cell; NASH, nonalcoholic steatohepatitis; NAFLD, nonalcoholic fatty liver disease; PSMP, PC3-secreted microprotein; -, no reported.

**Figure 1:**
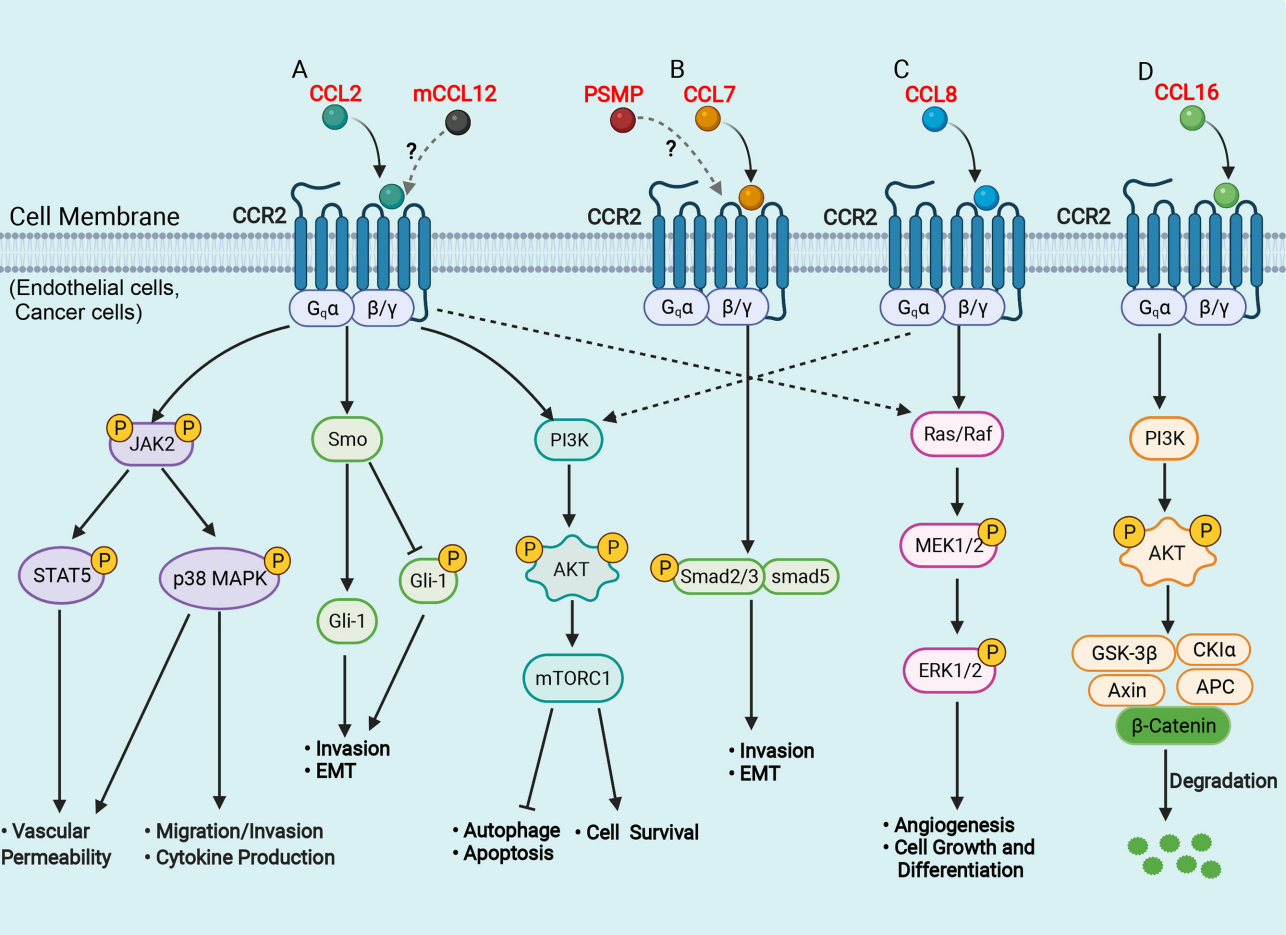
Schematic representation of signaling pathways in the liver of CCR2 and its ligands. **(A)** CCL2 binds to CCR2 and activates JAK2, triggering several downstream pathways, such as STAT5 and p38MAPK, which enhance vascular permeability, promote cell migration and invasion, favor cytokine production; CCL2 binds to CCR2 and activates the Hh signaling pathway, and increases the expression of Smo and Gli-1, which induce cell invasion and EMT; CCL2 binds to CCR2 and activates the PI3K/Akt/mTORC1 pathway, promoting cell survival and inhibiting autophagosome formation and cell apoptosis. **(B)** CCL7 binds to CCR2 and activates the TGF-β/Smad signaling pathway, which promotes cell invasion and EMT; **(C)** CCL8 binds to CCR2 and activates the Ras/Raf/MEK/ERK pathway, enhancing cell growth and differentiation and promoting angiogenesis. **(D)** CCL16 binds to CCR2 and activates PI3K/AKT/GSK3β signaling, which results in GSK3β proteasomal degradation and enhances β-catenin stability. AKT, protein kinase B; APC, adenomatous polyposis coli; CK1, casein kinase 1; EMT, epithelial-mesenchymal transition; ERK, extracellular signal-regulated kinase; Hh, hedgehog; Gli, glioblastoma; GSK3β, glycogen synthase kinase-3 beta; JAK2, Janus kinase 2; MAPK, mitogen-activated protein kinase; mTOR, mammalian target of rapamycin; MEK, mitogenic effector kinase; PI3K, phosphatidylinositol 3-kinase; Smo, smoothened; STAT, signal transducer and activator of transcription protein. (Figure created with BioRender.com).

From the perspective of the molecular mechanism, the binding of the chemokine receptor CCR2 with its ligands can cause subsequent intracellular signal transduction, in which the CCL2/CCR2 axis is the most studied and reported (**Figure 1A**). Tumor cell-derived CCL2 binds to its corresponding chemokine receptor CCR2 on endothelial cells, which in turn activates the Janus kinase 2 (JAK2)-signal transducer and activator of transcription protein 5 (STAT5) and p38 mitogen-activated protein kinase (MAPK) signaling pathways, resulting in enhanced vascular permeability and effective extravasation of tumor cells by recruitment of monocytes (41). CCL2 also promotes cancer cell migration and invasion by activating the p38 MAPK pathway, leading to tumor metastasis (42). The hedgehog (Hh) and TGF-β signaling pathways can regulate cell proliferation, activation, differentiation and participate in the pathogenesis of NASH, cirrhosis, and primary liver cancer (43, 44). CCL2 bound to CCR2 can induce HCC cell invasion and epithelial-mesenchymal transition (EMT) through activation of the Hh signaling pathways and increase the expression of Smo and glioblastoma 1(Gli-1) (14, 24). In addition, the CCL2/CCR2 axis can promote cell survival by inhibiting autophagic death and apoptosis of cancer cells by activating the phosphatidylinositol 3-kinase (PI3K)/AKT/mammalian target of rapamycin (mTOR)-dependent pathway (45, 46). CCL7 has been found to activate the TGF-β/Smad signaling pathway in HCC cells through CCR2 to promote HCC metastasis (24, 47) (**Figure 1B**). Furthermore, CCL8 can promote endothelial cell migration, proliferation, and angiogenesis through CCR2 by activating the extracellular signal-regulated kinase (ERK) 1/2 signaling pathway (48, 49) (**Figure 1C**). Another recent study has shown that CCL16 binds explicitly to CCR2, which in turn activates the AKT/mitogenic effector kinase (GSK3β) signaling and promotes β-catenin nuclear translocation, leading to enhanced cancer cell stemness (50) (**Figure 1D**).

Since there is no comprehensive and up-to-date review of the functions of all known chemokine ligands of CCR2 in liver disease, the purpose of this article is to summarize all the information about the involvement of each chemokine ligand for CCR2 in the development of liver disease. This information can provide a deeper understanding of the immune mechanism of chemokines involved in liver diseases. It may also help develop new therapeutic strategies targeting CCR2 and its ligands, especially for liver fibrosis/cirrhosis and HCC.

## CCL2 in Liver Disease

CCL2, also known as MCP-1, was the first CC chemokine discovered in humans. Murine CCL2, also known as JE, was originally a platelet-derived growth factor (PDGF)-inducible gene found in mouse 3T3 fibroblasts (33). The human version of CCL2 was purified from the human myelomonocytic cell line (THP-1) (34). CCL2 has apparent chemotactic effects on monocytes, T lymphocytes, natural killer (NK) cells, and dendritic cells (DCs) and is also an activator of basophils and mast cells (51, 52). Chemotaxis experiments showed that CCL2 could bind to receptors such as CCR1, CCR2, CCR4, and CCR11 based on its binding affinity (53–55). CCL2 can be secreted by different cell types, including monocytes, macrophages, DCs, endothelial cells, epithelial cells, fibroblasts, astrocytes, microglia, neurons, smooth muscle cells, keratinocytes, mesangial cells, and osteoblasts (51, 56, 57). CCL2 can be induced by a variety of mediators, including interleukins (IL)-1β and IL-4, tissue necrosis factor α (TNF-α), transforming growth factor β (TGF-β), interferon γ (IFN-γ), PDGF, vascular endothelial growth factor (VEGF), granulocyte-macrophage colony-stimulating factor (GM-CSF) and bacterial lipopolysaccharide (LPS) (56–58). In response to proinflammatory responses, such as LPS, TNF-α, or IL-1β, tumor cells produce CCL2 dependent on the activation of the constitutive nuclear factor -κB (NF-κB) pathway (57). Type I IFN receptor mediates the expression of CCL2 induced by modified vaccinia virus Ankara, which in turn recruits NK cells and T lymphocytes to the site of infection (58). The study has also found that chemokine CXCL12 treatment of endothelial cells can upregulate CCL2 expression through PI3K/Akt and p38 MAPK-dependent signaling pathways (59). In addition, the T helper type 2 (Th2) cytokines IL-4 and IL-13 can upregulate the expression of CCL2 in epithelial cells by activating p38 MAPK, ERK and JAK-2 signaling pathways (60).

**Figure 2:**
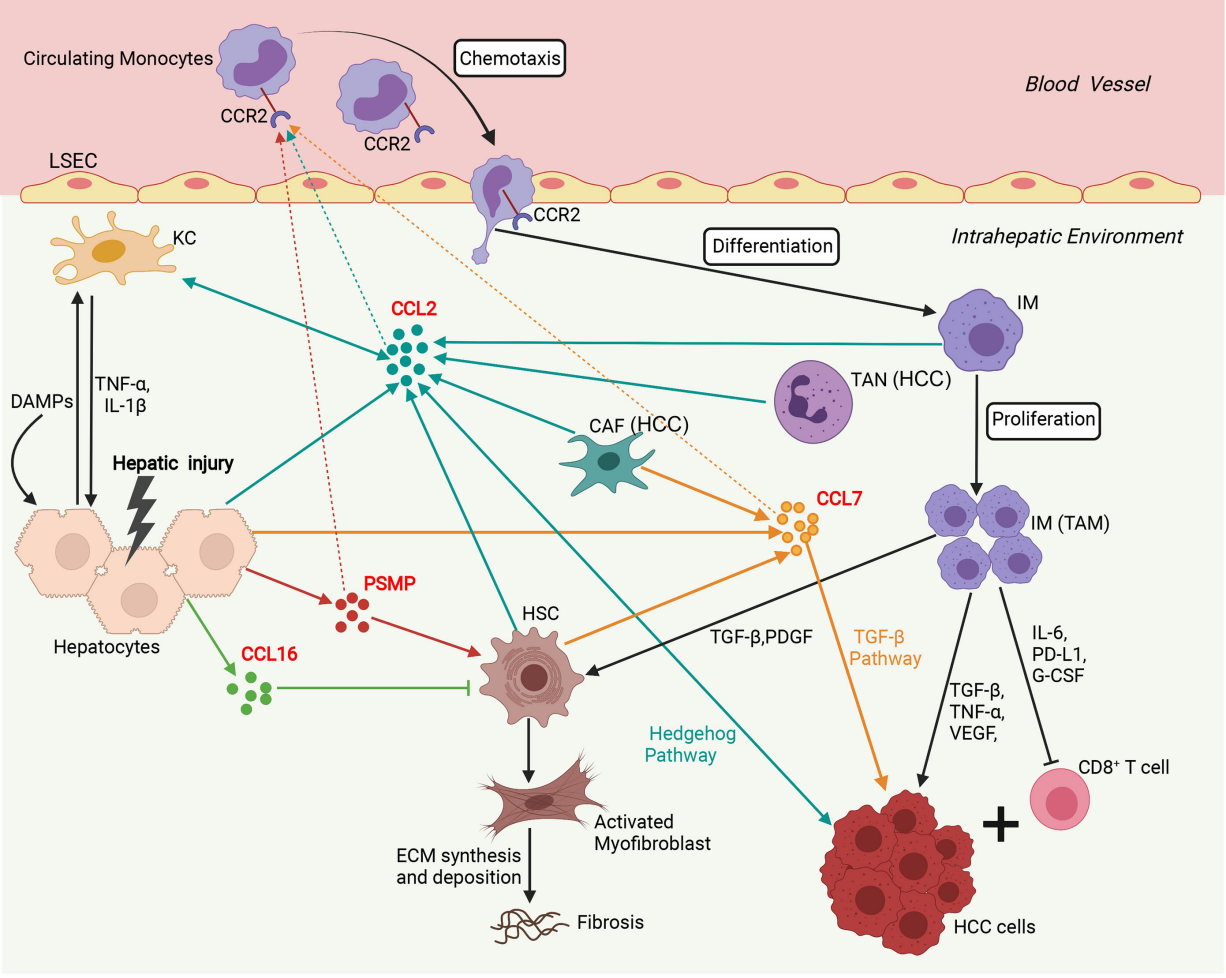
Involvement of the network of CCR2 and its ligands in regulation of immune mechanisms during liver injury, fibrosis and hepatocarcinogenesis. Sophisticated experimental mouse models of liver injury, fibrosis, and HCC revealed the complex interplay of different chemokines and liver cells. In the initial phase, upon hepatocyte injury, DAMPs activate KCs that in turn secrete inflammatory cytokines (TNF-α, IL-1β), which contribute to further hepatocyte injury and release chemokines including, CCL2, CCL7, CCL16, and PSMP. CCL2, CCL7, and PSMP promote the recruitment of CCR2^+^ circulating monocytes into the injured liver, where they develop into inflammatory, angiogenic, and fibrogenic IMs. During chronic injury, these macrophages activate HSCs by secreting TGF-β and PDGF to become collagen-producing myofibroblasts responsible for excessive extracellular matrix (ECM) synthesis and deposition, promoting liver fibrosis development. In addition, CCL16 directly inhibits the activation of HSCs, and PSMP directly promotes the activation of HSCs during liver fibrosis. In the HCC tumor microenvironment, TANs and CAFs could recruit TAMs by secreting CCL2 and CCL7. TAMs, specifically the M2 phenotype, promoted tumor growth, invasiveness, and metastasis by suppressing CD8^+^ T lymphocyte responses and producing PD-L1, IL-6, G-CSF, TNF-α, TGF-β, and VEGF. Additionally, CCL2 could directly promote HCC progression through activation of the Hh pathway. CCL7 could directly enhance the mesenchymal phenotype of HCC cells and facilitate their migration and invasion through the TGF-β signaling pathway. CAF, cancer-associated fibroblast; DAMP, danger-associated molecular pattern; ECM, extracellular matrix; G-CSF, granulocyte-colony stimulating factor; HCC, hepatocellular carcinoma; HSC, hepatic stellate cell; IM, infiltrating macrophage; KC, Kupffer cell; LSEC, liver sinusoidal endothelial cell; PDGF, platelet-derived growth factor; PD-L1, programmed death-ligand 1; PSMP, PC3-secreted microprotein; TAM, tumor-associated macrophage; TAN, tumor-associated neutrophil; TNF-α, tissue necrosis factor α; TGF-β, transforming growth factor β; VEGF, vascular endothelial growth factor. (Figure created with BioRender.com).

In addition to its chemotactic effect on immune cells, numerous studies have shown that CCL2 can mediate multiple biological functions, including cell activation, atherosclerosis, fibrosis, angiogenesis, and tumorigenesis (6, 61, 62). In the liver, CCL2 is a multifunctional regulator of liver pathology and modulates all stages of liver disease progression, from initial liver injury through inflammation and chronic hepatitis B virus (HBV)/hepatitis C virus (HCV) infection to fibrosis/cirrhosis and hepatocarcinogenesis (14–18). Additionally, CCL2 expression was significantly upregulated in liver cancers and correlated with the prognosis of HCC patients (13). We summarize the immune network of the CCL2/CCR2 axis that functions in liver disease in **Figure 2**. After the liver is damaged by viral infections, alcohol abuse, or metabolic disorders, liver cells, such as hepatocytes, Kupffer cells (KCs), and HSCs, secrete CCL2, which recruits many circulating CCR2^+^ monocytes to the injured liver (6–8, 15, 16). Next, these CCR2^+^ monocytes differentiate into infiltrating macrophages (IMs) with proinflammatory, angiogenic, and fibrogenic phenotypes, rapidly expanding the macrophage pool in the liver (6–8, 15). During chronic injury, hepatic IMs mediate the transdifferentiation of HSCs to become collagen-producing myofibroblasts by secreting TGF-β and PDGF (6–8, 15). Furthermore, pharmacologically inhibiting hepatic monocyte/macrophage infiltration by blocking CCL2 alleviates steatohepatitis and liver fibrosis in mice (17, 18). In the liver tumor microenvironment, a high infiltration density of TAMs, specifically the M2 phenotype, contributes to tumor progression and poor prognosis of patients with HCC (13, 62–64). Mechanistically, TAMs promote tumor growth, invasiveness, metastasis, and EMT by acting as myeloid-derived suppressor cells (MDSCs) that suppress CD8^+^ cytotoxic T lymphocyte (CTL) responses and by producing IL-6, programmed death-ligand 1 (PD-L1), granulocyte-colony stimulating factor (G-CSF), TNF-α, TGF-β and VEGF that accelerate HCC cell proliferation and invasion (13, 65–67). In addition, CCL2 can directly induce HCC cell invasion and EMT by activating Hedgehog signaling in a CCR2-dependent manner (14). CCL2 secreted by oncogene-induced senescent hepatocytes can recruit CCR2^+^ immature myeloid cells and then inhibit the antitumor function of NK cells, thereby promoting HCC tumor growth (19). In addition, tumor-associated neutrophils (TANs) can mediate the intratumoral infiltration of macrophages and regulatory T cells (Tregs) by secreting CCL2, which contributes to HCC progression, metastasis, and prognosis (20, 21).

Moreover, the expression of CCL2 is significantly upregulated in HCV-infected patients and is negatively correlated with the abundance of miR-122 in the liver, indicating that miR-122 may negatively regulate CCL2 (22, 23). miR-122 is the most abundant liver-specific microRNA (miRNA), accounting for approximately 52% and 70% of the total liver miRNA population in adult human and mouse, and plays a central role in diverse aspects of hepatic function and in liver pathology (68). Under physiological conditions, miR-122 is highly expressed in both human and mouse liver during embryonic development (68, 69). miR-122 is involved in liver development by targeting a group of genes to regulate hepatocyte proliferation, differentiation, maturation and polyploidy (69, 70). Under pathological conditions, miR-122 has been extensively investigated as a serum biomarker for the severity of hepatocyte damage which causes the release of miR-122 into the circulation (69, 70). Studies have found that miR-122 is significantly upregulated in the serum of NAFLD/NASH and alcoholic liver disease (ALD) patients (71, 72). Tang et al. reported that iron overload can significantly reduce the expression of miR-122, which binds to the 3’UTR of CCL2 mRNA and leads to increased CCL2 expression, and ultimately triggering the NF-κB-mediated inflammatory responses, such as IL-6, TNF-α, and IL-1β expression (73). Therefore, in addition to the quantitative evaluation of HCV, the determination of miR-122 and CCL2 should also be considered in predicting the development and progression of HCV-mediated HCC.

Collectively, these studies have confirmed the essential role of the chemotactic cytokine CCL2 in the progression of liver disease, especially in the development of HCC. Pharmacological blockade of the CCL2/CCR2 signaling could significantly reduce the inflammatory response and inhibit chronic liver disease progression, suggesting that the CCL2/CCR2 axis may be a promising therapeutic target for treating human liver diseases, especially the prevention of the progression of HCC.

## CCL7 in Liver Disease

CCL7, also known as MCP-3, was a chemotactic cytokine first discovered in the supernatants of human osteosarcoma cells (MG-63) (35). Human CCL7 showed 73% identity with human CCL2 by amino acid sequence alignments (35) (**Table 2**). CCL7 can be expressed in cell types such as immune cells, stromal cells, and airway epithelial cells and can also be expressed in various tumor cells under pathological conditions (35, 79). Through its functional receptors (CCR1, CCR2, CCR3, and CCR5), CCL7 can effectively chemoattract various immune cells, including monocytes/macrophages, T lymphocytes, NK cells, DCs, neutrophils, and eosinophils (74, 79, 80).

**Table 2:** Amino acid sequence alignment of human CCL2, CCL7, CCL8, CCL13, CCL16, PSMP, mouse CCL12, and their homologies with CCL2, binding affinities to CCR2.

Chemokine	Amino acid sequences	Identities with CCL2 (%)	Affinities to CCR2 (K_d_)	Refs
CCL2	*MKVSAALLCLLLIAATFIPQGLA* QPDAINAPVT	100	~0.5 nM	(34, 53)
	**CC**YNFTNRKISVQRLASYRRITSSK**C**PKEAVIFK			
	TIVAKEICADPKQKWVQDSMDHLDKQTQTPKT			
CCL7	*MKASAALLCLLLTAAAFSPQGLA* QPVGINTSTT	73	~13 nM	(35, 74)
	**CC**YRFINKKIPKQRLESYRRTTSSH**C**PREAVIFK			
	TKLDKEICADPTQKWVQDFMKHLDKKTQTPKL			
CCL8	*MKVSAALLCLLLMAATFSPQGLA* QPDSVSIPIT	69	3±1 nM	(35, 75)
	**CC**FNVINRKIPIQRLESYTRITNIQ**C**PKEAVIFKT			
	KRGKEVCADPKERWVRDSMKHLDQIFQNLKP			
mCCL12	*MKISTLLCLLLIATTISPQVLA* GPDAVSTPVT	68	~5 nM	(36, 76)
	**CC**YNVVKQKIHVRKLKSYRRITSSQ**C**PREA			
	VIFRTILDKEICADPKEKWVKNSINHLDKTS			
	QTFILEPSCLG			
CCL13	*MKVSAVLLCLLLMTAA* FNPQGLAQPDALNVPST	65	~15 nM	(37, 77)
	**CC**FTFSSKKISLQRLKSYVITTSR**C**PQKAVIFRTKLGKEICADPKEKWVQNYMKHLGRKAHTLKT			
CCL16	*MKVSEAALSLLVLILIITSASRS* QPKVPEWVNTPST	31	~95 nM	(38, 78)
	**CC**LKYYEKVLPRRLVVGYRKALN**C**HLPAIIFVTKRNREVCTNPNDDWVQEYIKDPNLPLLPTRNLSTVKIITAKNGQPQLLNSQ			
PSMP	*MALRMLWAGQAKGILGGWGIICLVMSLLLQHPGVYS* KCYFQAQAPCHYEGKYFTLGESWLRKDCFHCTCLHPVGVG**CC**DTSQHPIDFPAG**C**EVRQEAGTCQFSLVQKSDPRLPCKGGGPDPEWGSANTPVPGAPAPHSS	–	~1.4 nM	(40)

The sequence data of each ligand of CCR2 is avaibable from GenBank, respectively. Conserved cysteine residues are indicated by the red font; N-terminal signal peptide sequence of the mature proteins are indicated by the blue font.

According to reports, CCL7 has played different and even opposite roles in various inflammatory diseases. CCL7 could promote mouse colitis models by chemotactic macrophages and acute LPS-induced lung inflammation by recruiting neutrophils (81, 82). Additionally, CCL7 could inhibit cutaneous inflammation by limiting the accumulation of neutrophils (83). CCL7 was also associated with the development of a variety of tumors. The expression of CCL7 was significantly increased in gastric cancer, renal cancer, and pancreatic ductal adenocarcinomas (84–86). Compared with primary colorectal cancer (CRC), the expression of CCL7 in liver metastases of CRC was significantly increased, suggesting CCL7 as a novel target with potential clinical value in preventing CRC liver metastases (87).

A recent study showed that in the liver, CCL7 was significantly upregulated in an LPS-induced mouse model of acute liver injury and a methionine-and-choline-deficient (MCD) diet-induced mouse model of chronic liver injury, accompanied by increased macrophage infiltration (25). Further studies showed that CCL7 could also be induced in hepatocytes treated with LPS or palmitate (25). The chromatin remodeling protein Brahma-related gene 1 (BRG1) interacts with activator protein 1 (AP-1) to activate the transcription of CCL7, which in turn promotes macrophage accumulation in the liver (25). In addition, microarray analysis showed that consecutive ERK1/2 activation induces the expression of senescence related secretory factors including CCL7, indicating that CCL7 can be used as a marker of human HSC senescence (26). In the liver tumor microenvironment, there is a group of specialized fibroblasts that are defined as cancer-associated fibroblasts (CAFs) (88, 89). CAFs can directly promote the proliferation of HCC cells, increase angiogenesis, and secrete chemokines to recruit immune cells to promote tumor growth and metastasis, and they are closely related to the poor prognosis of HCC patients (90, 91). Liu et al. reported that CCL7 secreted by CAFs enhanced the EMT of HCC cells and promoted their migration and invasion through the TGF-β pathway (24).

**Figure 3:**
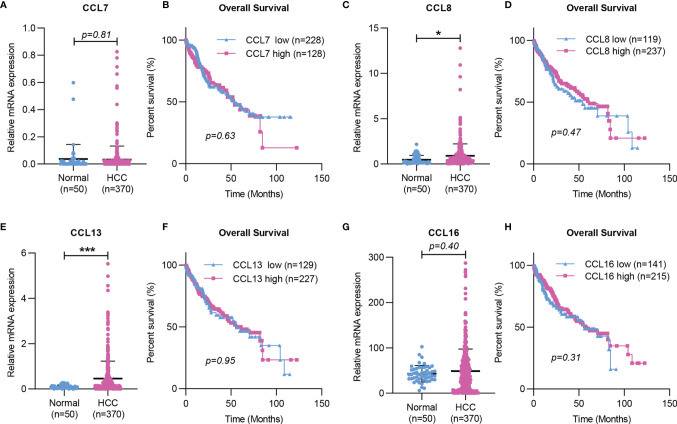
Expression and Kaplan-Meier survival curves of CCL7, CCL8, CCL13, and CCL16 in HCC based on TCGA. **(A, C, E, G)** The mRNA expression of CCL7, CCL8, CCL13, and CCL16 in HCC tissues was determined by RNA-seq data from the TCGA LIHC dataset. **(B, D, F, H)** The correlation of CCL7, CCL8, CCL13, and CCL16 mRNA expression levels with overall survival time was determined by RNA-seq in the TCGA LIHC dataset. TCGA, The Cancer Genome Atlas. (*P < 0.05; ***P < 0.001).

Taken together, the working model of CCL7 in liver disease is summarized in **Figure 2**. During liver injury, damaged hepatocytes or senescent HSCs can promote macrophage infiltration in the liver and lead to the progression of hepatic inflammation by producing CCL7. In the tumor microenvironment, CAFs can promote HCC metastasis by secreting CCL7 to mediate EMT. To further explore the potential role of CCL7 in HCC, gene expression data of CCL7 in HCC were obtained from the liver hepatocellular carcinoma (LIHC) database of the Cancer Genome Atlas (TCGA). There was no significant difference in the expression level of CCL7 between HCC tumor tissues and adjacent tissues (**Figure 3A**). In addition, we divided the HCC cases into low-expression (CCL7 low) and high-expression (CCL7 high) groups according to the mRNA level of CCL7 and investigated the correlation of CCL7 expression with the prognosis of HCC patients. The expression level of CCL7 has no significant correlation with the prognosis of HCC patients (**Figure 3B**).

These findings indicate that, on the one hand, inhibitors/antagonists of CCL7 can be screened to inhibit the progression of hepatitis. On the other hand, CCL7 can be used as a potential therapeutic target for HCC metastasis, although further studies are needed.

## CCL8 in Liver Disease

CCL8, also known as MCP-2, is another chemotactic cytokine found in the supernatant of human osteosarcoma cells (MG-63) (35). CCL8 shares 69% structural homology with CCL2 and exerts a chemotactic effect on monocytes, T lymphocytes, NK cells, basophils, and eosinophils (35, 92, 93) (**Table 2**). CCL8 can bind to several chemokine receptors, including CCR1, CCR2, CCR3, CCR5, and CCR11 (55, 75, 94, 95). Previous studies demonstrated that CCL8 was secreted by monocytes/macrophages, fibroblasts, endometrial cells, and mast cells (35, 94). CCL8 can also be produced by CD169^+^ bone marrow derived macrophages (BMDMs) in response to stimulation with LPS and high-mobility group box 1 (HMGB-1) (96). In addition, hypoxia in cervical cancer cells increases the expression of Zinc finger E-box binding homeobox 1 (ZEB1), which directly upregulates the production of CCL8 and then recruits macrophages (97).

Previous studies have demonstrated that CCL8, as a chemokine, could regulate the recruitment of immune cells in different tissues in inflammatory diseases. In a mouse model of chronic atopic dermatitis, CCL8 could drive chronic eosinophilic inflammation by mediating the accumulation of CD4^+^ Th2 cells in the skin through CCR8 (98). CCL8 could promote dextran sulfate sodium (DSS)-induced ulcerative colitis in mice by recruiting inflammatory monocyte-derived macrophages and is a potential target for treating mucosal damage (96). In addition, CCL8 was involved in the migration, invasion, and stemness of cancer cells in the tumor microenvironment. Chen et al. reported that CCL8 mediates the infiltration of TAMs through the CCR2-NF-κB pathway and participates in the progression and development of cervical cancer (97). CCL8 secreted by TAMs recruits Tregs through CCR5, promoting tumor cell migration and invasion, leading to lung cancer metastasis (99).

In the liver, CCL8 expression was significantly increased in patients with spontaneous clearance of HCV compared with chronic HCV-infected patients (27). CCL8 was also found to be increased in NASH *in vitro* models (28). In addition, Helier et al. reported that an amino acid change in CCL8, Q46K, was closely related to the severity of liver fibrosis in HCV-infected patients (29). Compared with adjacent tissues, the expression level of CCL8 was significantly upregulated in HCC tumor tissues based on the TCGA LIHC dataset (**Figure 3C**). However, the expression level of CCL8 was not associated with the prognosis of HCC patients (**Figure 3D**).

Although no published studies have shown the specific role of CCL8 in liver disease, combined with the essential role CCL8 plays in other inflammatory diseases and tumor processes suggests that CCL8 may be involved in hepatitis virus infection, liver inflammation, steatosis, or fibrosis.

## CCL12 in Liver Disease

Murine CCL12 (mCCL12), also known as murine MCP-5, is a chemokine that was initially identified in the lungs during allergic inflammation (36, 76, 100). The mature mCCL12 protein is 68% identical to the mature human CCL2 protein (76, 100) (**Table 2**). mCCL12 can exert potent chemotactic effects on monocytes/macrophages, T and B lymphocytes, and eosinophils through its only known chemokine receptor CCR2 (36, 76, 100). mCCL12 is mainly expressed by stromal cells in lymph nodes, and its expression is significantly increased in activated macrophages (36, 76, 100).

Elevated mCCL12 can promote the development of lung fibrosis induced by fluorescein isothiocyanate by chemotaxis of fibroblasts in mice (101). In addition, Yang et al. reported that mCCL12 produced by injured alveolar epithelial cells could promote pulmonary fibrosis in mice by recruiting profibrotic macrophages through CCR2 in mice (102). The increased peripheral level of mCCL12 in old mice can aggravate brain injury induced by intracerebral hemorrhage by recruiting macrophages and T cells (103). In the tumor microenvironment, mCCL12 has been reported to play an essential role in tumor progression by regulating the recruitment and polarization of macrophages. The upregulation of mCCL12 expression in the premetastatic lungs can enhance tumor cell arrest and metastasis by recruiting monocytic MDSCs (mo-MDSCs) (104). In irradiated MC38 and LLC tumor models, IFN-β can induce mCCL12 expression by activating the stimulator of interferon genes (STING) pathway, which in turn mobilizes CCR2^+^ Mo-MDSCs into tumors, leading to radioresistance (105). In addition, GM-CSF released by murine breast cancer 4T1 cells markedly upregulated the production of mCCL12 by peritoneal inflammatory macrophages, which in turn recruited monocytes to shape the tumor microenvironment (106).

To date, no further reports have been made on the role of mCCL12 in liver disease, with the exception of a longitudinal analysis that found that male mice, a chronically increasing trend in mCCL12 was associated with the progression of NAFLD to HCC (30). However, based on the high homology of mCCL12 with the human CCL2 protein (68%), mCCL12 may also play an essential role in liver pathology, which needs to be confirmed by further studies.

## CCL13 in Liver Disease

Human CCL13, also known as MCP4, was first isolated from a human heart cDNA library using a human eotaxin genomic probe. At the amino acid level, human CCL13 has 65% identity with human CCL2 (37) (**Table 2**). CCL13 acts as a chemoattractant for monocytes/macrophages, T lymphocytes, immature DCs, eosinophils, and basophils by binding to CCR1, CCR2, CCR3, CCR5, and CCR11 (37, 55, 95, 107). In addition to its chemotactic function, CCL13 can cause basophils to release histamine and eosinophils to degranulate, and it can cause the expression of adhesion molecules and the production of proinflammatory cytokines in endothelial, epithelial, and muscle cells (108, 109). CCL13 is widely expressed by different tissues under homeostasis conditions and is significantly increased in various tumor cell lines (A549, BEAS-2B) (77, 110). When epithelial cells are stimulated by cytokines (TNF-α, IL-1β and IFN-γ) or pathogen-associated molecular patterns (PAMPs) through Toll-like receptors (TLRs), the transcription factor NF-κB is subsequently activated, leading to the release of CCL13 along with other chemokines (77, 110). In addition, IL-4 markedly inhibited CCL13 expression induced by TNF-α and IL-1β in peripheral blood mononuclear cells but had little effect on CCL13 expression in epithelial cells (111).

Studies have shown that CCL13 is upregulated in asthma and allergic rhinitis and is associated with the number of monocytes/macrophages and eosinophils recruited in the airway of patients (112, 113). The expression of CCL13 was significantly increased in patients with chronic atopic dermatitis (AD), and its expression level was closely related to the number of CD68^+^ macrophages in AD skin lesions (114). In addition, CCL13 could trigger immune-modulatory responses by regulating the functions of muscle, epithelial and endothelial cells (115). These results suggested that CCL13 plays an essential role in various chronic inflammations by regulating immune cell infiltration. However, there is no published research showing the importance of CCL13 in liver disease. The mRNA level of CCL13 was significantly higher in HCC tumor tissues than in HCC-adjacent tissues based on the TCGA dataset (**Figure 3E**). No significant correlation was found between CCL13 expression and the clinical prognosis of HCC patients (**Figure 3F**). In addition, CCL13 has been found to lead to drug resistance in tumor cells by promoting cell apoptosis and drug resistance (116). Since CCL13 can bind to several chemokine receptors (CCR1, CCR2, CCR3, CCR5, or CCR11), it should have similar properties as other ligands of these receptors in the liver disease, but further studies are needed.

## CCL16 in Liver Disease

CCL16, also reported as liver-expressed chemokine (LEC), lymphocyte and monocyte chemoattractant (LMC), human CC chemokine (HCC)-4, and liver-specific CC chemokine (LCC)-1, is a human CC chemokine that was initially discovered based on the GenBank EST database (38, 117, 118). CCL16 is constitutively expressed by liver parenchymal cells and could also be expressed by monocytes treated with IL-10, IFN-γ, and LPS (117, 118). Shen et al. demonstrated that IL-10 mediates STAT3 activation, which then binds to the CCL16 promoter gene and enhances its expression, thereby increasing cancer cell stemness (50). Mature human CCL16 protein contains 120 amino acids and shows 25%-31% identity to other human CC chemokines (CCL2, CCL7, CCL8, CCL13) (117, 118) (**Table 2**). CCL16 has chemotactic effects on monocytes and T lymphocytes through its low-affinity receptors: CCR1, CCR2, and CCR5 (78, 119).

Under physiological conditions, human plasma contained a relatively high concentration of CCL16, which may regulate immune homeostasis (78). CCL16 has been reported to be upregulated in the serum of patients with pneumonia, irritable bowel syndrome patients with diarrhea, and LPS-induced WI-38 cells (120, 121). CCL16 can also trigger the angiogenic program in vascular endothelial cells through CCR1, which may play a role in tumorigenesis (122, 123). CCL16 was upregulated in breast tumors and closely correlated with tumor progression (50, 124). In addition, reports have shown that CCL16 can also mediate antitumor immunity by enhancing the effector and antigen-presenting cell (APC) function of macrophages and can augment T cell lytic activity by inducing the overexpression of TNF-α and Fas ligand (FasL) (125). Furthermore, CCL16 exhibited potent myelosuppressive activity and markedly induced tumor rejection by promoting APC-T cell cross-talk (126).

Recent studies have shown that CCL16 expression is downregulated in LO2 after LPS and hypoxia stimulation (31). CCL16 could reduce liver cirrhosis by inhibiting the activation of HSCs in mice, suggesting that it may be a marker to predict the occurrence and progression of liver cirrhosis (31). There was no significant difference in the expression level of CCL16 between HCC tumor tissues and HCC-adjacent tissues, and the expression level of CCL16 had no correlation with the prognosis of HCC patients based on the TCGA LIHC dataset (**Figures 3G, H**).

These studies suggest that, unlike other chemokine ligands of CCR2, CCL16 is a negative regulator in liver diseases. It may limit the progression of liver fibrosis/cirrhosis by inhibiting the activation of HSCs. In addition, CCL16 may play a role in the HCC process by promoting T cell-mediated antitumor activity, which needs to be confirmed by further studies.

## PSMP in Liver Disease

PSMP is a newly identified chemotactic cytokine that acts as a CCR2 ligand and was initially found in the prostate cancer cell line PC3 and prostate cancer tissues (39, 40). PSMP can chemoattract peripheral blood monocytes and lymphocytes through CCR2, and the affinity of PSMP and CCR2 is equivalent to that of CCL2 and CCR2 (40) (**Table 2**). Several studies have shown that PSMP may play an essential role in inflammation and tumor progression. Pei et al. reported that PSMP could promote DSS-induced colitis in mice by chemoattracting Ly6C^hi^ monocytes through CCR2 (127). Another study found that PSMP secreted by ovarian cancer cells induced by hypoxia could promote angiogenesis through the MAPK signaling pathway (128). In addition, the expression level of PSMP in patients with chronic active antibody-mediated rejection (CAAMR) has been enormously increased, and there is a significant correlation with the number of infiltrating CD68^+^ macrophages (129). We found that IL-1β, damage-associated molecule pattern (DAMP) molecules IL-33 and HMGB-1 could induce mouse primary hepatocytes to produce PSMP (32). PSMP could recruit inflammatory macrophages through CCR2 to infiltrate and promote M2-type polarization of macrophages and directly activate HSCs, ultimately promoting the progression of liver fibrosis (32). Moreover, the expression of PSMP was markedly increased in human cirrhotic and HCC-adjacent liver tissues (32).

These results suggest that PSMP may play an essential role in other liver diseases by regulating immune cells and HSCs, warrants further investigation.

## Discussion

**Figure 4:**
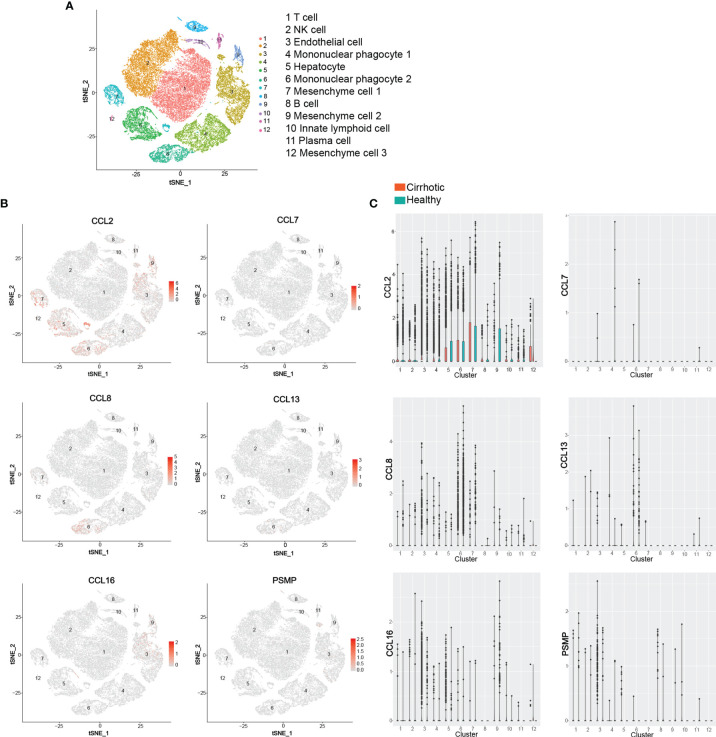
ScRNA-seq analysis of human liver. **(A)** t-SNE projection showing a reference map of 12 liver cell clusters with established cell-specific marker genes in human liver. **(B, C)** t-SNE plots showing relative distribution of CCL2, CCL7, CCL8, CCL13, CCL16 and PSMP in the 12 identified cell clusters in human healthy and cirrhotic liver and its statistical summary. scRNA-seq: Single-cell RNA sequencing; t-SNE: t-distributed stochastic neighbor embedding.

Chemokines and chemokine receptors are considered important mediators of innate immune cell transport to address acute inflammation and are crucial factors that determine the immune cell fate in adaptive immune responses. In recent years, extensive *in vivo* and *in vitro* studies have shown that the chemokine receptor CCR2 and its ligand system play an essential role in the pathogenesis of various acute and chronic liver diseases, including the regulation of liver homeostasis inflammation, fibrosis, and cancer. Based on the public database of liver single cell RNA sequencing (ScRNA-seq), we identified 12 liver cell clusters (T cell, NK cell, Endothelial cell, Mononuclear phagocyte 1, Hepatocyte, Mononuclear phagocyte 2, Mesenchyme cell 1, B cell, Mesenchyme cell 2, Innate lymphoid cell, Plasma cell, Mesenchyme cell 3) in the liver by established cell-specific marker genes (130, 131) (**Figure 4A**). ScRNA-seq analysis of human liver showed that hepatocyte, mononuclear phagocyte (macrophage and KC), mesenchyme cell are the main cell sources of CCL2 in both human healthy and cirrhotic liver; CCL7 is almost undetectable in any cell clusters in human liver; Mononuclear phagocytes (macrophage and KC) are the major source of CCL8 and CCL13 in both human healthy and cirrhotic liver; Endothelial cells (LSEC) and hepatocytes are the major source of CCL16, especially in human cirrhotic liver; Endothelial cells (LSEC) are the major source of PSMP under physiological conditions (**Figures 4B, C**). However, the specific functions of various CCR2 chemokine ligands in liver diseases are not entirely consistent. In this review, we examined the reported evidence of the role of each CCR2 chemokine ligand in different stages of liver disease.

In the liver, parenchymal cells, such as hepatocytes, and nonparenchymal cells, such as HSC and LSEC, directly act as the main sensors and triggers of the immune response (132, 133). In addition, the liver contains a large number of immune cells (KCs, monocytes, DCs, and T cells) that are involved in maintaining homeostasis (132, 133). KCs are resident and self-renewing phagocytes in the liver, which can recognize, ingest and degrade cellular debris, foreign substances or pathogens, serving as the key sentinels for liver homeostasis (133, 134). As an important part of the reticuloendothelial system (also known as the mononuclear phagocyte system), resident KCs interact with hepatic parenchymal cells, nonparenchymal cells and other immune cells to form a highly active and dynamic complex network that regulates liver immune homeostasis (135). KCs usually constitutively express distinct scavenger receptors, complement receptors, and pattern recognition receptors, so they can be activated in response to both infectious and non-infectious stimuli to induce immunogenic T cell responses (133, 136). In addition, the ingestion of particulate antigens results in antigen-processing by KCs and induction of IL-10-expressing Tregs, thereby mediating the establishment of tissue protective systemic tolerance (133, 136). Furthermore, low-level exposure to endotoxins from the gut microbiota induces ‘endotoxin tolerance’, which leads to active immune suppression through tolerogenic factors such as IL-10, TGF-β and prostaglandin E (PGE2) expressed by KCs and DCs (137).

Given the critical roles of CCR2 chemokine ligands in liver homeostasis and disease, the regulation of their expression has also been a focus of research in the past decade. Upstream regulators include a variety of inflammatory cytokines such as TNF, IL-6, IL-1β and IFN-γ (138). Among them, TNF induces NF-κB signaling by activating cis-regulatory elements such as proximal promoters and distal super-enhancers, and activates gene expression in a context-dependent manner (139, 140). During liver injury, TNF-α stimulation of LSECs induces p300 interaction with NF-κB and the epigenetic reader protein bromodomain containing 4 (BRD4) and acetylation of lysine-27 on histone-3 (H3K27) at CCL2 enhancer and promoter regions to activate CCL2 gene transcription (141). In addition to NF-κB, other transcription factors, such as AP-1, ZEB1 and STAT3, are also involved in stimulating the transcription of chemokines induced by the aforementioned chemokines. BRG1 interacts with AP-1 to regulate CCL7 transcription in a redox-sensitive manner mediated by BRG1 phosphorylation catalyzed by casein kinase 2 (CK2) in hepatocytes (25). Hypoxia in the tumor microenvironment can promote the expression of transcription factor ZEB1, thereby upregulating CCL8 production by directly binding to the CCL8 promoter region (97). In addition, increased DNA damage can enhance the DNA sensing pathway through cyclic GMP-AMP synthase (cGAS)/STING activation, promoting type I interferon production and signal transduction, leading to the induction of chemokines including CCL2, CCL7 and mCCL12 (105). The promoter region of the CCL13 gene also has consensus sequences for the transcription factors NF-κB, AP-2 and glucocorticoid receptors (115). Furthermore, IL-10 treatment promotes expression of STAT3 phosphorylation, mediates STAT3 activation, which then binds to the CCL16 promoter gene and enhances its expression (50). There is no report about transcriptional regulation on PSMP, and further study is needed.

Primary liver cancer is the sixth most commonly diagnosed cancer and the third leading cause of cancer death worldwide in 2020, with approximately 906,000 new cases and 830,000 deaths (142, 143). HCC is the most common form of primary liver cancer (comprising 75%-85% of cases) and most frequently develops on a background of chronic liver disease and cirrhosis (143, 144). Although our understanding of the pathogenesis of HCC has improved, this knowledge has not been fully translated into clinical practice (145–147). Treatment strategies for HCC are increasing, including improvements in ablation techniques, and local and systemic treatments. However, the morbidity and mortality of HCC remain high, and the long-term prognosis is still unsatisfactory (145–147). Therefore, it is necessary to explore new strategies to treat HCC. The development and clinical trials of effective drugs for CCR2 and its ligands are underway. These studies may further enrich the treatment strategies for chronic liver disease and even liver cancer.

The CCL2-CCR2 axis is currently the most well-studied chemokine pathway in liver disease. CCR2 and its ligands have become therapeutic targets of interest for biopharmaceutical companies. Among them, CVC is an orally active, dual antagonist of CCR2 and CCR5 (9). Preclinical and clinical studies indicate that CVC is an effective and safe antifibrotic agent for treating alcoholic steatohepatitis and NASH with fibrosis, and it is currently being tested in phase III clinical trials (10–12). In addition, CCL2 has proinflammatory, profibrotic, and proangiogenic effects in chronic liver disease and tumor-promoting effects in HCC, making it a potential drug target for the treatment of liver disease (13, 14, 16–18, 22, 23). Studies have shown that in mouse chronic liver injury models, mNOX-E36, a novel structured L-enantiomeric RNA oligonucleotide, could significantly reduce the infiltration of Ly-6C^hi^ monocytes and abnormal angiogenesis, thereby accelerating the regression of liver fibrosis (17, 18, 148).

Our previous studies revealed that PSMP is a novel CCR2 high-affinity ligand and its function *in vivo* and *in vitro* is dependent on the CCR2 (32, 40, 127). The affinity between PSMP and CCR2 was found to be comparable to that between CCL2 and CCR2 (40). For clinical applications, PSMP may be a safer and more specific new therapeutic target: 1. PSMP is lowly or not expressed in most normal tissues and is significantly elevated in a variety of human disease and mouse models (32, 40, 127). Compared with wild-type mice, PSMP knockout mice showed no obvious abnormalities (127). While CCR2 is constitutively expressed in various normal tissues, and impeding the CCR2 signaling prevents normal egress of monocytes from bone marrow under physiological conditions (149); 2. In the mouse models of liver fibrosis, the effects of blocking PSMP on disease evaluation indicators are similar to those of blocking CCR2, indicating that PSMP is the major ligand for CCR2 to play a key role in the live pathological process (32); 3. PSMP is produced by parenchymal cells (e.g., colon epithelial cells, hepatocytes) of the injured tissue, rather than by immune cells, to recruit pro-inflammatory monocytes/macrophages into the injured tissue and initiate a series of downstream immune responses (32, 127). CCL2 has been considered to be the major ligand of CCR2. However, no significant expression of CCL2 was detected in parenchymal cells of the injured tissue (32). During the inflammation process, infiltrating activated monocytes/macrophages produce a variety of pro-inflammatory cytokines, including CCL2 (56–58). Our previous studies have shown that blocking PSMP can significantly reduce the levels of pro-inflammatory cytokines (IL-6, TNF-α, and CCL2), indicating that PSMP, as a key upstream molecule, plays a regulatory role in inflammation (32, 127). Taken together, combined with the low expression of PSMP in most normal tissues and upregulation in damage-induced injured tissues, targeting PSMP for liver disease may have long-term therapeutic safety and tolerability.

miRNAs are small (20-24 nucleotide), non-coding RNAs that regulate gene expression and cellular processes by binding to specific mRNA targets and promoting their degradation and/or translational inhibition (150). Hepatic miRNAs profiles play an important role in the pathogenesis of liver diseases by regulating liver metabolism, injury, fibrosis and tumor development (151). Based on the obvious relevance in liver pathophysiology, miRNAs have been proposed as attractive targets for the diagnosis and treatment of liver diseases (68–72). Currently, many miRNAs have been or are being evaluated in preclinical studies or clinical trials for their potential as biomarkers for liver disease diagnosis, prognosis, and treatment response. For example, the ratio of miR-122-5p/miR-151a-5p has been identified as a reliable marker for predicting postoperative liver dysfunction in patients with liver malignancies undergoing hepatectomy (152). Teufel et al. have identified nine plasma miRNAs, including miR-122, as biomarkers that can predict regorafenib response in patients with HCC (153). In addition, clinical studies have shown that exosomal miRNAs, such as miR-122 and miR-21, are expected to be used for the early detection and prediction of HCC (154). In the future, more basic and clinical research should be performed to further explore and validate the superiority and utility of miRNAs in predicting, diagnosis, monitoring, and treatment of liver disease.

In conclusion, the vital role of the chemokine ligand CCL2 in liver diseases makes it a potential therapeutic target for chronic liver disease and even HCC. However, other ligands of CCR2 have not been thoroughly studied in liver diseases, especially CCL7, CCL8, CCL12, CCL13, CCL16, and PSMP. The role of these chemokines in other inflammatory diseases and tumors has been widely reported. On the one hand, they can act as chemokines to recruit corresponding immune cells to inflammatory or tumor sites; on the other hand, they can act directly on target cells or tumor cells, thereby promoting or inhibiting the progression of diseases. In addition, the protein structures these chemokines are highly homologous (25%-73%). Therefore, targeting chemokine receptor CCR2 and its ligands for treating chronic liver disease and HCC has excellent prospects, especially in combination with immunotherapy, and further studies in preclinical animal models and liver disease patients’ clinical data are required.

## Author Contributions

Conception and design of the study, SS and HC. Data collection, analysis, and interpretation, SS, LR, and PC. ScRNA-seq analysis, MW. Drafting of the manuscript, SS. Critical revision of the paper, DC, YW, and HC. Approval of the final draft, SS, LR, PC, MW, DC, YW, and HC. All authors contributed to the article and approved the submitted version.

## Funding

This project was supported by the National Key Sci-Tech Special Project of China (No. 2018ZX10302207, No. 2017ZX10203202) and the China Postdoctoral Science Foundation (No. 2021M690260).

## Conflict of Interest

The authors declare that the research was conducted in the absence of any commercial or financial relationships that could be construed as a potential conflict of interest.

## Publisher’s Note

All claims expressed in this article are solely those of the authors and do not necessarily represent those of their affiliated organizations, or those of the publisher, the editors and the reviewers. Any product that may be evaluated in this article, or claim that may be made by its manufacturer, is not guaranteed or endorsed by the publisher.
